# Network Pharmacology and Molecular Docking Approach to Reveal the Immunotherapeutic Mechanism of Cuscutae Semen in Treating Thin Endometrium

**DOI:** 10.1155/2022/4333128

**Published:** 2022-09-22

**Authors:** Wenyan Zhang, Yuan Yuan, Guangrong Huang

**Affiliations:** Department of Gynaecology, Shenzhen Bao'an Chinese Medicine Hospital, Guangzhou University of Chinese Medicine, Shenzhen, China

## Abstract

**Objective:**

Thin endometrium is considered as a leading cause of infertility, recurrent pregnancy loss, and repeated implantation failure. The seed of Cuscutae Semen (CS) has been used to prevent aging and improve sexual function in Traditional Chinese Medicine. However, the pharmacological mechanism of CS in preventing and treating thin endometrium remains to be elucidated.

**Methods:**

Three public databases, TCMSP, GeneCards, and OMIM, were searched to collect the main active compounds and putative molecules of CS, as well as the targets of thin endometrium, respectively. The CS and thin endometrium common targets were subject to protein-protein interaction (PPI) analysis followed by functional enrichment analysis. The best binding mode of CS compounds and common target proteins was evaluated by molecular docking and analysis in the AutoDockTools.

**Results:**

In total, 11 main active compounds, 102 drug target proteins, and 70 CS and thin endometrium common targets were identified. There were 68 nodes with 722 edges in the PPI network; HIF1A, MYC, ESR1, and EGFR were the top 4 targets. After functional enrichment analysis, it was revealed that the therapeutic effects of active compounds of CS on thin endometrium were achieved through cellular response to chemical stress, transcription regulator, DNA-binding transcription factor binding, chemical carcinogenesis-receptor activation, lipid, and atherosclerosis. The molecular docking analysis revealed that the 3 active compounds of CS, quercetin, matrine, and isorhamnetin, have good binding ability with their targets, HIF1A, MYC, ESR1, and EGFR.

**Conclusion:**

Our study uncovers the main active compounds in CS and their corresponding targets related to thin endometrium which explains the pharmacological mechanism underlying therapeutic effects of CS on thin endometrium.

## 1. Introduction

The human endometrium is the inner lining of the uterus that is a highly regenerative tissue at each cycle under estrogen stimulation in the menstrual cycle [1]. Its sole purpose is to enable embryo implantation and pregnancy. Successful pregnancy depends on a receptive endometrium of adequate thickness concomitant with a well-developed embryo [2]. The proliferative phase of the menstrual cycle where the endometrium experiences cyclic proliferative and secretory changes is very important for endometrial growth [3]. Thin endometrium is defined as insufficient endometrial thickness with a maximum thickness not more than 7 mm on an ultrasound in the middle luteal phase (i.e., 6-10 days after ovulation), which is considered as a leading cause of recurrent miscarriage and poor embryo implantation, thus resulting in long-term infertility [4, 5]. More specifically, thin endometrium was found to be an independent risk factor of hypertensive disorders of pregnancy in a study based on 13,458 patients in frozen-thawed embryo transfers [6]. The pathogenesis of thin endometrium refers to low estrogen level, insufficient progesterone level, ovulation disorder, and lack of growth hormone [7]. In addition to those, thin endometrium may result from high blood flow resistance of uterine arteries, uterine fibroids, and uterine malformations, as well as inappropriate endometrium repair following many times of curettage and surgical separation of intrauterine adhesion [8]. Recently, stem cell-based therapy by various administration approaches has gain massive attention in the regeneration of thin or damaged endometrium [9]. However, the resident, differentiation, and survival potential of administered stem cells remain formidable challenges to develop the best route of administration for stem cell-based therapy. In addition to cell therapy, autologous platelet-rich plasma could serve as a noninvasive front-line therapy to enhance endometrial thickness and improve implantation in patients with recurrent implantation failure and thin endometrium [10]. Traditional Chinese Medicine (TCM) exhibits significant advantages in the treatment of female diseases including thin endometrium due to their pharmacological properties of improving uterine blood flow and inhibiting platelet aggregation [11, 12].

Cuscutae Semen (CS), originated from the dry and mature seeds of Cuscuta australis R. Br. or Cuscuta chinensis Lam., has been widely used as Chinese medicine since ancient [13]. CS can nourish the liver and kidney, prevent miscarriages, and protect the eyesight due to its immunomodulatory function and antioxidant effect [14]. Besides, CS exerts an immunosuppressive effect on dendritic cells, and its active ingredient kaempferol could reduce cytokines and chemokines released by dendritic cells stimulated by lipopolysaccharide, indicating the therapeutic potential of CS in chronic inflammatory and autoimmune diseases [15]. Multiple studies reported the therapeutic effects of mesenchymal stem cells by improving the endometrium thickness probably via their migration and immunomodulatory properties [16, 17]. CS can promote the proliferation and growth of stem cells [18]. Besides, CS and Fructus Lycii can effectively inhibit spermatogenic cell apoptosis and promote their proliferation [19]. However, the pharmacological mechanism of CS in preventing and treating thin endometrium remains to be elucidated.

**Figure 1:**
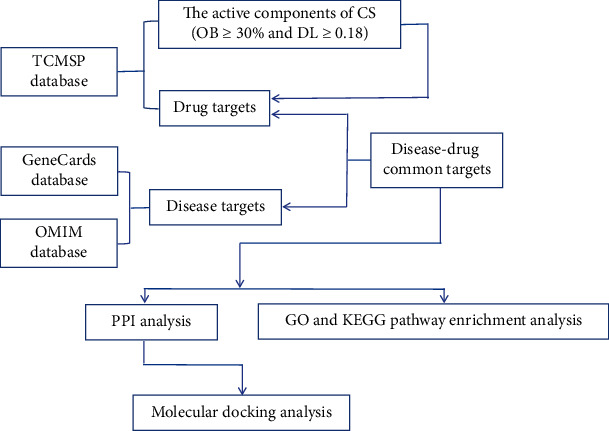
A workflow presenting the study design.

The dominant paradigm in drug discovery refers to the concept of designing ligands with maximum selectivity to act on individual drug targets [20]. However, the pharmacological actions of many effective drugs were achieved by modulating multiple proteins not just a single target. In 2008, Hopkins proposed the concept of network pharmacology which elaborates a new paradigm to systematically reveal the mechanisms behind drug therapy on human diseases at the whole organismal level [21]. As system biology and bioinformatics tools develop, the strategy of network pharmacology shifted into “drug-target-pathway-disease” rather than “one drug, one target”, which can offer a more integrative analysis of TCM mechanisms [22]. Molecular docking is an important method for drug discovery in structural molecular biology and computer-assisted drug design, which can not only simulates the geometric structure of molecules and but also estimate the best binding modes of two interacting molecules [23].This study performed a network pharmacology approach with the aid of molecular docking technology (Figure 1) to decipher the mechanism elucidating the preventive effects of CS against thin endometrium.

## 2. Methods

### 2.1. Active Compounds and Action Targets of CS

The Traditional Chinese Medicine Systems Pharmacology Database and Analysis Platform (TCMSP) is a public pharmacology platform covering Chinese herbal medicines that captures the relationships between drugs, targets, and diseases [24]. We used this platform to obtain the active components of CS. In light of 30% oral bioavailability (OB) and 0.18 drug-likeness (DL) were the lowest level to evaluate pharmacokinetic actions of the compounds of herbal medicines, the active components of CS with OB ≥ 30% and DL ≥ 0.18 in the TCMSP are regarded as the main active components. The proteins (only “Homo sapiens”) corresponding to the above active components were transformed into gene symbols using the UniProt database [25].

### 2.2. Common Target Mining

The target genes related to thin endometrium were acquired from two public databases: the GeneCards database (https://www.genecards.org/) and Online Mendelian Inheritance in Man database (OMIM, https://omim.org/). Duplicate values were removed. The Venn diagram of the thin endometrium-associated targets and the targets related to the active components of CS was made using the R software to obtain disease-drug common targets. The disease-target-compound network was visualized using the Cytoscape software to visualize.

### 2.3. Protein-Protein Interaction (PPI) Analysis

The disease-drug common targets were mapped into the STRING 11.0 database (https://www.string-db.org/) to obtain the PPI network which was visualized by importing the tsv-based file to the Cytoscape software (3.8.1). The species must be “Homo sapiens,” and high confidence for interaction score must not less than 0.4. In the PPI network, nodes reflect proteins, and connecting lines represent PPIs. The core genes ranked according to degree value obtained using cytoHubba plug-in of Cytoscape.

### 2.4. Functional Classification and Pathway Enrichment

Gene Ontology (GO) functional analysis and KEGG-based pathway analysis were implemented to harvest the potential functions of the disease-drug common targets by using the “clusterProfiler” in the R software. The results of GO analysis were presented at three levels: biological processes, molecular functions, and cellular components. KEGG stores the items linking metabolic pathways or signal transduction pathways. The GO terms at three levels and significant KEGG pathways enrichments were ranked by the value of GeneRatio, and the top 20 pathways and top 10 GO functions were presented as bubble plots using the “pathview” package in the R software.

### 2.5. Molecular Docking Technology

Molecular docking technology is a well-recognized method to examine receptor-ligand interactions along with binding patterns and affinities. Therefore, we performed molecular docking analysis between curcuma and the top core target genes in the PPI network. The pdb format of the 3D structure of the proteins encoded by the top core target genes was downloaded from the RCSB Protein Data Bank (PDB) database, accessed at https://www.rcsb.org/. Then, we converted the pdb-based files containing curcuma and the proteins encoded by core targets into pdbqt-based files and search for active pockets. The AutoDockTools was employed to determine the binding ability of ligands and receptors. The binding energy less than 0 indicates spontaneous binding of ligand and receptor, and smaller values reflect higher binding activity.

## 3. Results

### 3.1. The Main Active Compounds and Druggable Targets of CS

First, we searched the active compound components of CS in the TCMSP database and collected active ones using an OB at least 30% and DL at least 0.18 as cutoff values; a total of 11 active compounds of CS were identified (Table 1). We removed duplicate druggable targets of CS, converted these protein names into gene symbols, and obtained 102 target genes, in total, of the active compounds of CS in the TCMSP database.

### 3.2. Identification of CS and Thin Endometrium Common Targets

**Figure 2:**
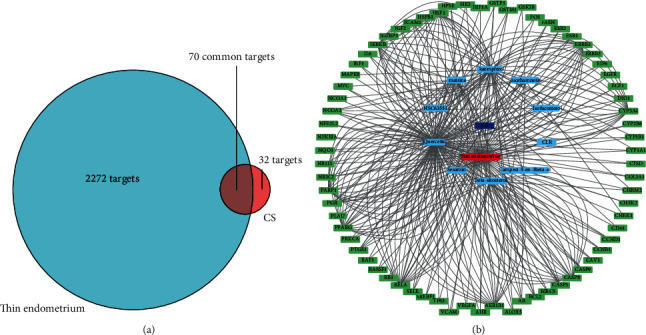
(a) Venn diagram of 70 druggable targets of CS which were also therapeutic targets of thin endometrium. (b) Disease-target-compound network based on the 10 bioactive compounds of CS and the 70 thin endometrium-CS common targets.

**Figure 3:**
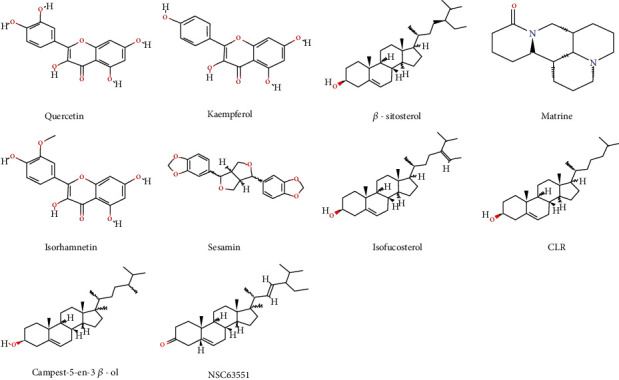
The structure of 10 bioactive compounds of CS.

Next, we searched the GeneCards and OMIM databases and sorted out 2342 targets of thin endometrium, respectively. Accordingly, we mapped 2342 thin endometrium-related disease targets to 102 target genes of the active compounds of CS by using Venny 2.1 drawing software. We finally identified 70 CS and thin endometrium common targets (Figure 2(a)). We then used the Cytoscape software to present disease-target-compound network with a total of 82 nodes based on one disease, one drug, 10 active monomers of CS, and the 70 common targets (Figure 2(b)), suggesting the multicomponent and multitarget characteristics of CS. The 10 bioactive compounds were matrine, sesamin, isorhamnetin, quercetin, isofucosterol, kaempferol, NSC63551, CLR, campest-5-en-3*β*-ol, and *β*-sitosterol.  Figure 3 shows the structure of 10 bioactive compounds of CS.

### 3.3. PPI Network Construction

**Figure 4:**
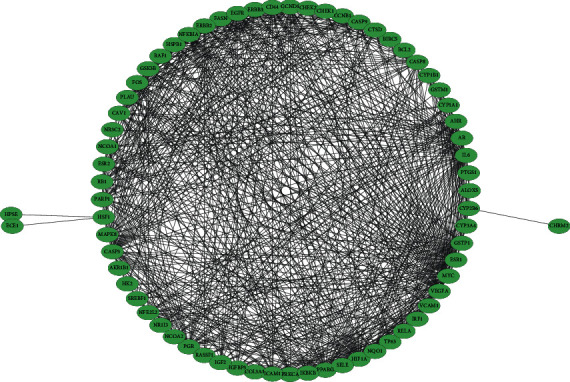
PPI network construction for 70 CS and thin endometrium common targets.

We introduced 70 CS and thin endometrium common targets into the STRING database for PPI analysis. As presented by Figure 4, there were 68 nodes with 722 edges in the PPI network, and those with higher degree values were regarded as corer target genes. Degree values of targets ranged from 1 to 49. HIF1A, MYC, ESR1, and EGFR were the top 4 targets, with degree values of 49, 49, 48, and 46, respectively. Among 70 common targets, DIO1 and EIF6 were excluded out of the PPI network due to weak interaction.

### 3.4. Enrichment Analysis for Thin Endometrium-CS Common Targets

**Figure 5:**
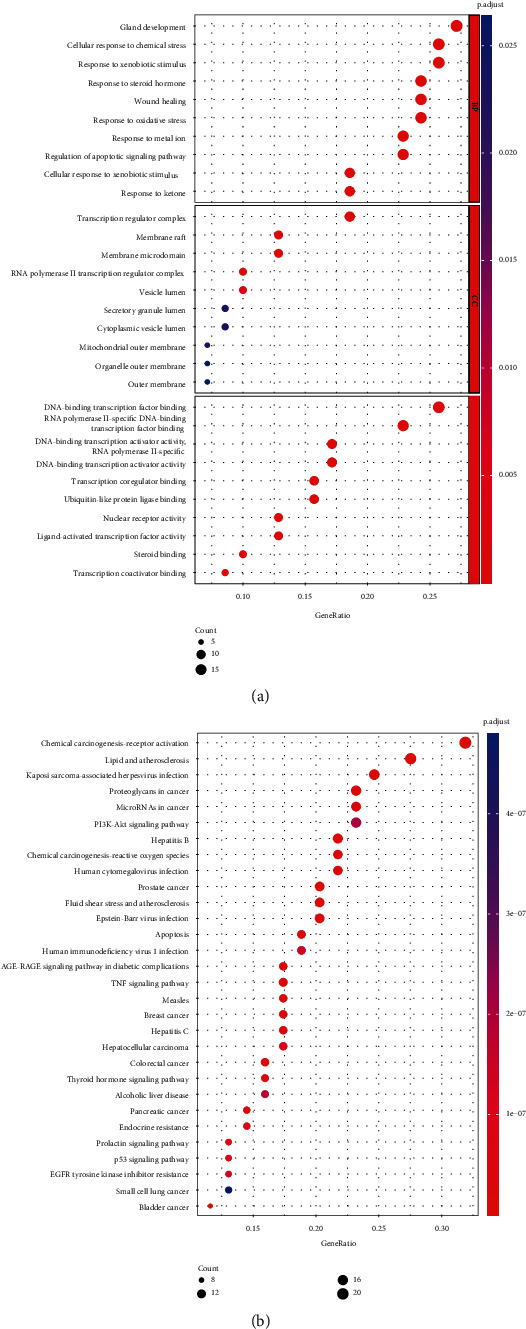
The top 10 most enriched GO terms at the levels of BP, CC, and MF (a) and the top 20 most enriched KEGG pathways (b), presented by bubble plots.

Next, we further analyzed 70 CS and thin endometrium common targets by GO annotation and KEGG pathway analyses. After GO analysis, a total of 1243 GO terms were identified to be significantly enriched by these thin endometrium-CS common targets (*p* < 0.05). The active ingredients of CS were associated with 1146 terms at the level of BP, 15 terms at the level of CC, and 82 terms at the level of MF.  Figure 5(a) lists the top 10 most enriched GO terms at the levels of BP, CC, and MF. After KEGG pathway analysis, we found that 131 KEGG pathways were significantly enriched by these disease-drug common targets (*p* < 0.05).  Figure 5(b) lists the top 20 most enriched KEGG pathways.

### 3.5. Molecular Docking and Analysis

**Figure 6:**
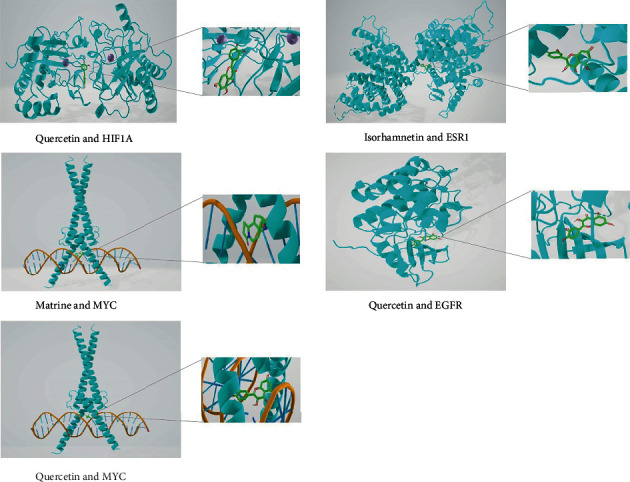
Molecular docking analysis of quercetin and HIF1A, matrine and MYC, quercetin and MYC, isorhamnetin and ESR1, and quercetin and EGFR.

HIF1A, MYC, ESR1, and EGFR as top 4 targets in the core PPI network were selected for molecular docking and analysis. The affinity energy ≤ −5 kcal/mol is considered as high affinity. The binding energy between quercetin and HIF1A (-8.90 kcal/mol), matrine and MYC (-8.40 kcal/mol), quercetin and MYC (-8.50 kcal/mol), isorhamnetin and ESR1 (-7.80 kcal/mol), and quercetin and EGFR (-7.80 kcal/mol) suggests that the 3 active compounds of CS, quercetin, matrine, and isorhamnetin, have good binding ability with their targets, HIF1A, MYC, ESR1, and EGFR (Figure 6).

## 4. Discussion

It is reported that adequate uterine receptivity and good quality of endometrium are necessary factors for successful pregnancy. Thin endometrium (endometrial thickness < 6 mm) may be a contributing factor to the increased risk of infertility [26]. TCM appears to be an effective method in the treatment of female endometrium disease, such as thin endometrium [11], dysfunctional uterine bleeding accompanied by endometrial hyperplasia [27], and low endometrial receptivity [28]. As a Chinese medical material, CS (the ripe dried seeds of Cuscuta australis R.Br. and C. chinensis Lam.) has been widely used to prevent aging, weakness of the loins and knees, osteoporosis [29], and ease pain and inflammation [30]. However, the underlying mechanism and efficacy of CS in thin endometrium remain to be explored.

Network pharmacology-based approach combined with molecular docking has gained popularity in exploring active compounds of TCM, key targets acting on diseases, and potential pharmacological mechanism of TCM against diseases [31–33]. On the basis of three public databases, TCMSP, GeneCards, and OMIM, the present study obtained 11 active compounds of CS, 102 target genes of the active compounds of CS, and 2342 targets of thin endometrium. Subsequently, 70 CS and thin endometrium common targets, and 10 active monomers of CS including matrine, sesamin, isorhamnetin, quercetin, isofucosterol, kaempferol, NSC63551, CLR, campest-5-en-3*β*-ol, and *β*-sitosterol were obtained. Lastly, the top 4 key targets including HIF1A, MYC, ESR1, and EGFR were identified according to the PPI network. Hypoxia plays an important role in in the pathology of human diseases represented by cancers [34], inflammatory bowel disease [35], and neurological disorders [36]. Hypoxia represents an important microenvironment condition, which contributes to drug resistance in cancers through regulation of signal pathways [37]. Endometrium is a kind of multicellular tissue, and there may be hypoxia in endometrium during menstruation and implantation [38]. The transcriptional activation of a series of genes involved in cell proliferation and angiogenesis was conducive to the survival of hypoxia-exposed cell. HIF1 is oxygen regulated transcription activator and participates in the process of cell proliferation, angiogenesis, and inflammation. In vitro culture of human primary term cytotrophoblasts. Overexpressed HIF1A mRNA in peripheral blood mononuclear cells is related to poor overall survival of chronic lymphocytic leukemia [39]. Females with preeclampsia showed elevated HIF1A mRNA expression in placenta, which was 3-fold higher than that of control women [40]. MYC gene consists of C-MYC, N-MYC, and L-MYC, which acts as a potential anticancer target [41]. Although there were no direct evidences showing correlations between MYC and thin endometrium, previous studies identified its role in endometrium related disease. For instance, higher mean percentage of cells expressing C-MYC was observed in endometrial carcinomas compared to endometrial hyperplasias and cyclic endometria [42]. In addition, the expression of C-MYC mRNA in proliferative endometrium was 65% higher than that in normal tissues [43]. Estrogen is involved in endometrial growth in primates. ESR signaling is the key regulator to maintain successful pregnancy, and the expression levels of ESR1 and ESR2 have impacts on endometrial receptivity [44]. Studies have shown that the women with ESR1 and ESR2 gene variants are more likely to experience unexplained recurrent pregnancy loss [45]. In a baboon model of endometriosis, decreased ESR1 level was showed in endometrial stromal cells, while reduced ESR-2 expression was displayed in endometrial stromal and glandular epithelial cells [46]. EGFR is a transmembrane glycoprotein which belongs to the ERBB family of tyrosine kinase receptors. The binding of EGFR and its ligand will lead to autophosphorylation of receptor tyrosine kinase and then activate the cascade downstream signal pathways that regulate cell proliferation, differentiation, division, and survival [47]. Suh et al. indicted that the activation of PI3K/AKT and PI3K/AKT through leucine-rich repeat and immunoglobulin-like domain 2 contributed to suppress the growth of Hec-1A endometrial cancer cells [48]. In our study, we further identified that 3 active compounds of CS, quercetin, matrine, and isorhamnetin, have good binding ability with these four key targets. Quercetin is a natural polyphenol compound, which exists in many Chinese medicinal materials. Pharmacological studies have shown that quercetin has property of anti-inflammatory, antioxidant, and immunomodulatory, and its mechanism may be related to nuclear factor-*κ*B inhibition and protein kinase B phosphorylation [49, 50]. Matrine has a wide range of biological characteristics including anti-inflammatory activity, anticancer activity, and antifibrosis activity [51]. Matrine reduced the expression of TNF-*α* and IL-1*β* and attenuated the uterus injury in the lipoteichoic acid-induced mouse endometritis model [52]. Isorhamnetin is involved in regulation of signaling pathways such as PI3K/AKT/PKB, NF-*κ*B, and MAPK to display antitumor, anti-inflammatory, and antioxidation prosperity [53].

Several limitations should be noticed when our results were interpreted. First, experimental validation in vivo and in vitro focusing on the suppressive effects of main 3 active compounds of CS, quercetin, matrine, and isorhamnetin, cells as well as the expressions of HIF1A, MYC, ESR1, and EGFR in the setting of thin endometrium is warranted to improve the preliminary nature of the study. Second, the molecular mechanism by which CS against thin endometrium is not completely characterized as these public databases we used in the study have been updated continuously.

In summary, the study demonstrates that CS, especially its main 3 active compounds of CS, quercetin, matrine, and isorhamnetin, may exert therapeutic effects on thin endometrium through the modulation of multiple targets, HIF1A, MYC, ESR1, and EGFR. The present work not only investigates the complex mechanism of CS in the treatment of thin endometrium with multicomponent, multitarget, and multisignal but also supports that network pharmacology prediction method with molecular docking verification may provide a preliminary but systemic exploration focusing on the pharmacokinetic properties and mechanism of TCM in human diseases, offering opportunities to develop micelles for targeted delivery of TCM to thin endometrium.

## Figures and Tables

**Table 1:** The basic information of these 11 active compounds in CS in the TCMSP database.

Mol ID	Molecule name	MW	OB (%)	DL
MOL005944	Matrine	248.41	63.77	0.25
MOL001558	Sesamin	354.38	56.55	0.83
MOL006649	Sophranol	264.41	55.42	0.28
MOL000354	Isorhamnetin	316.28	49.60	0.31
MOL000098	Quercetin	302.25	46.43	0.28
MOL005440	Isofucosterol	412.77	43.78	0.76
MOL000422	Kaempferol	286.25	41.88	0.24
MOL000184	NSC63551	412.77	39.25	0.76
MOL000953	CLR	386.73	37.87	0.68
MOL005043	Campest-5-en-3*β*-ol	400.76	37.58	0.71
MOL000358	*β*-Sitosterol	414.79	36.91	0.75

## Data Availability

The data used to support the findings of this study are included in the article.
